# Macro and micronutrient based soil fertility zonation using fuzzy logic and geospatial techniques

**DOI:** 10.1038/s41598-025-12184-3

**Published:** 2025-07-23

**Authors:** Meeniga Venkateswarlu, Srinivas Rallapalli, Amit Singh, G. Sai Sesha Chalapathi, Suresh Kumar, Yashwant Bhaskar Katpatal, Gouligari Sujatha

**Affiliations:** 1 Department of Civil Engineering, Birla Institute of Technology and Science, Pilani, Rajasthan India; 2 Department of Mechanical Engineering, Birla Institute of Technology and Science, Pilani, Rajasthan India; 3 Department of Electrical and Electronics Engineering, Birla Institute of Technology and Science, Pilani, Rajasthan India; 4 Agriculture, Forestry and Ecology Group, Indian Institute of Remote Sensing, Dehradun, India; 5 Department of Civil Engineering, Visvesvaraya National Institute of Technology, Nagpur, India; 6 National Remote Sensing Centre, Hyderabad, India; 7 School of Interdisciplinary Research and Entrepreneurship, Birla Institute of Technology and Science, Pilani, Rajasthan, India

**Keywords:** Crop productivity, Fuzzy logic, GIS, Soil fertility, Zone mapping, Environmental sciences, Chemistry, Engineering

## Abstract

Modeling the spatial variability and uncertainty of soil fertility parameters is crucial for sustainable agriculture but remains a challenge due to complex interactions between soil properties. Traditional models often assess individual parameters, such as pH or nitrogen (N), without considering their combined influence and uncertainty. This study develops a fuzzy logic and geoinformatics-based approach to simultaneously assess multiple soil fertility parameters. The model integrates 80 fuzzy rules to evaluate macro- and micronutrients, incorporating 250 soil samples analyzed using the PUSA Soil Test and Fertilizer Recommendation Meter (STFR). Experimental results showed soil fertility parameter ranges: pH (7.46–8.26), ECe (0.267–0.807 dS m^−1^), organic carbon (0.24–0.56%), N (85.56–146.32 kg ha^−1^), P (21.99–34.28 kg ha^−1^), K (116.41–156.16 kg ha^−1^), S (5.60–20.86 mg kg^−1^), Fe (1.065–5.095 mg kg^−1^), Mn (2.058–2.637 mg kg^−1^), Zn (0.748–1.105 mg kg^−1^), B (0.372–0.530 mg kg^−1^), and Cu (0.230–0.788 mg kg^−1^). The fuzzy model-derived fertility scores ranged from 41.55 to 52.60, with pH, organic carbon, nitrogen, phosphorus, potassium, and iron as critical parameters influencing fertility. Geostatistical kriging interpolation estimated fertility values at unsampled locations, generating a continuous, high-resolution soil fertility map for precision agriculture. Validation with crop yield data ranked suitability as: Pearl millet (0.919) > Mustard (0.890) > Wheat (0.863) > Barley (0.861). Multi-criteria decision analysis confirmed pearl millet as the most suitable crop based on fertility and yield potential. The study categorizes soil into low and moderate fertility zones across Jhunjhunu, Rajasthan, ensuring a systematic assessment for optimal nutrient management. By integrating fuzzy logic with GIS-based spatial modeling, this study enhances soil fertility classification, site-specific nutrient recommendations, and sustainable crop planning, reinforcing the role of fuzzy-GIS frameworks in precision agriculture.

## Introduction

Soil fertility is a crucial factor in determining the productivity and sustainability of agricultural systems. It refers to the soil’s ability to supply nutrients in adequate amounts and proper balance for optimal plant growth^1–3^. Key fertility indicators such as pH, organic carbon, electrical conductivity, and the availability of macronutrients (N, P, K) and micronutrients (Fe, Mn, Zn, Cu, B) play a pivotal role in influencing crop yield and quality^4–6^. Balancing soil fertility is essential for enhancing agricultural productivity, minimizing costs, and ensuring environmental sustainability^7^. The evaluation of soil fertility often involves dealing with uncertainties due to spatial variation, heterogenous nature and the nonlinear interactions of multiple soil parameters^8^.

Fuzzy logic, an advanced computational approach is designed to handle uncertainty and imprecision in data and provides an effective way for addressing these challenges by integrating diverse soil fertility indicators into a single framework^9,10^. Unlike traditional binary logic, which categorizes data into absolute true or false values, fuzzy logic allows for degrees of membership function, providing a more nuanced analysis of complex systems having uncertainty^9^. Fuzzy logic-based techniques like fuzzy AHP, fuzzy TOPSIS, fuzzy fault tree, fuzzy expert systems have been widely applied in various fields, including environmental science and agriculture, for its ability to model complex relationships and generate precise outputs from imprecise inputs^10,11^. By applying rule-based decision-making, fuzzy inference systems (FIS translate imprecise soil data into clear fertility assessments, enabling accurate soil productivity zoning and informed agricultural decision-making^12^. FIS tackles uncertainty by using membership functions to represent range of input data, such as pH, EC, organic carbon, and nutrient levels, as degrees of change rather than fixed values, allowing for more nuanced data interpretation. This approach accommodates the inherent variability in soil properties, providing a flexible framework for modelling. The integration of fuzzy rules allows for dynamic adjustments based on varying soil conditions, enhancing the effectiveness of nutrient management strategies.

Although, fuzzy based techniques have been widely applied in soil fertility evaluation to address the inherent uncertainties in soil data, there are certain limitations^13,14^. One significant drawback is the narrow focus on specific soil parameters, such as sodium, potassium, and calcium, while neglecting the comprehensive and simultaneous assessment of macro-micronutrients and fertility factors^15–17^. Additionally, many studies are geographically constrained, as their findings are based on specific experimental sites, such as the INCAPER farm in Espírito Santo, Brazil^18^. Sometimes simplified rule bases in the models failed to capture the complex, nonlinear relationships among multiple soil properties, resulting in less accurate evaluations^19^. Lastly, most fuzzy logic applications were region-specific, making them difficult to generalize across different agro ecological zones or cropping systems^20^. Many fuzzy logic models lacked integration with geospatial tools like Geographic Information Systems (GIS), which are crucial for generating spatially explicit fertility maps, and rarely accounted for temporal variability, limiting their dynamic applicability^21,22^. Furthermore, validation of these models against real-world experimental agricultural outcomes was often insufficient, raising concerns about their reliability^23^.

Kriging, a geostatistical interpolation technique enhances the precision of soil fertility assessments by effectively modeling spatial variability. By utilizing both the spatial coordinates and the statistical properties of sampled data, kriging provides more accurate and reliable predictions of soil properties across unsampled locations, thereby facilitating informed decision-making in soil management and agricultural practices^24^. Kriging enhances soil fertility mapping by predicting soil properties in unsampled areas based on spatial autocorrelation, improving the accuracy of fertility zoning and site-specific management^25,26^. This technique minimizes prediction errors, ensuring reliable assessments for precision agriculture.

This study integrates kriging with fuzzy logic to enhance soil fertility mapping by interpolating spatially continuous soil properties and incorporating expert knowledge for uncertainty management^27^. Kriging predicts fertility values in unsampled areas, while fuzzy logic classifies these predictions into meaningful fertility categories, ensuring precision in soil assessment and decision-making^28^. This hybrid approach improves spatial accuracy and supports site-specific nutrient management strategies. The novelty of the study includes investigation of a comprehensive set of soil parameters, including both macro and micronutrients and modeling them into FIS that uses expert-defined rules coupled with geospatial kriging interpolation, offering a holistic and adaptable framework for soil fertility assessment. In particular, the aims of the study were: (a) to develop a Fuzzy Inference System (FIS) model for soil fertility assessment by integrating fertility indicators, macronutrients and micronutrients based on experimental data obtained from PUSA STFR Meter, optimizing resource use and reducing costs for farmers. (b) generate and validate soil fertility maps using kriging technique to provide farmers with precise, location-specific insights and (c) conducting real-world testing of the model outputs and fertility maps by evaluating their relationship with the productivity of a selected crop, ensuring practical relevance and effectiveness in improving agricultural outcomes.

## Methodology

**Fig. 1:**
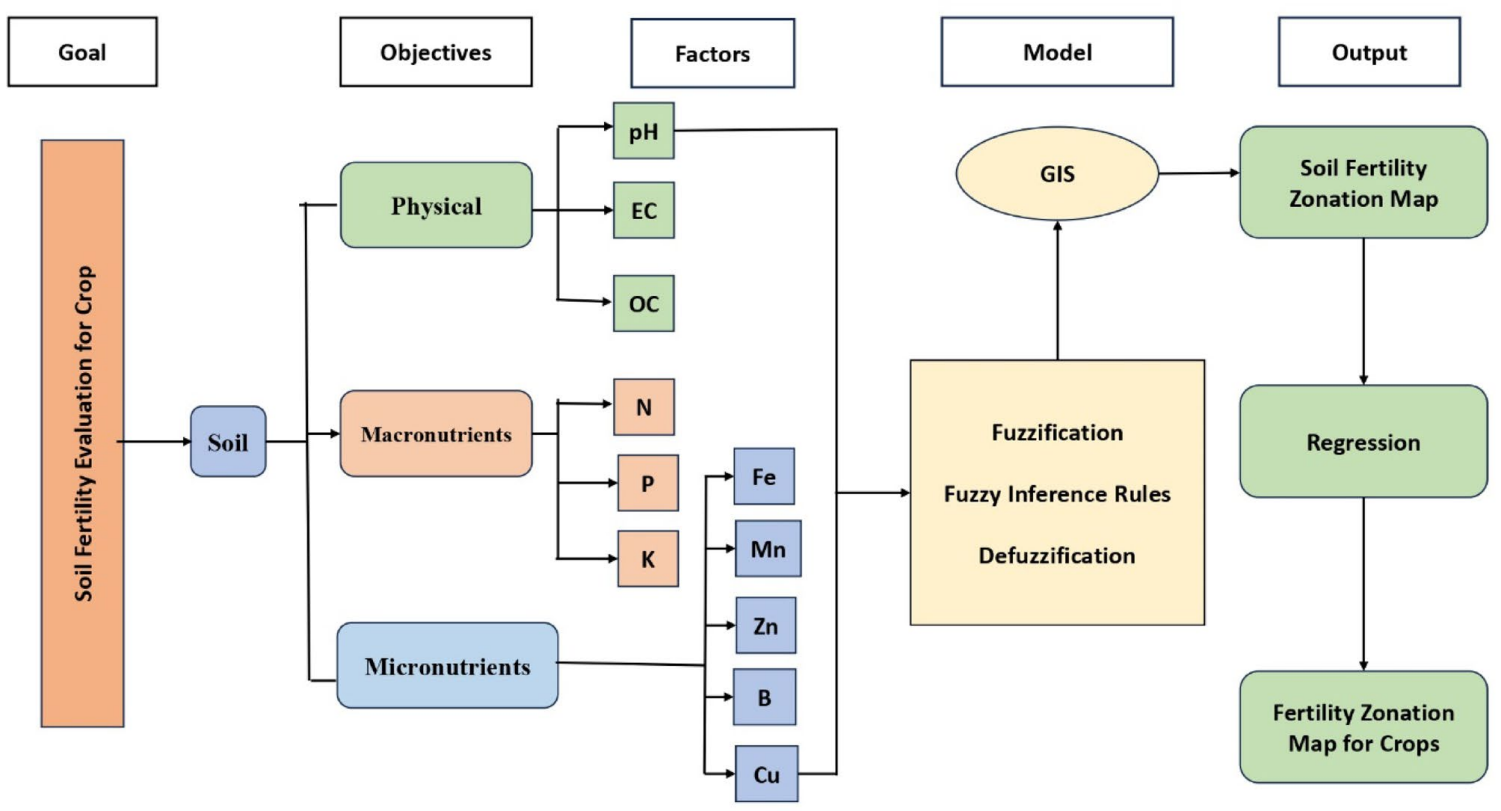
Schematic diagram of the FIS model for preparing soil fertility zonation.

**Fig. 2:**
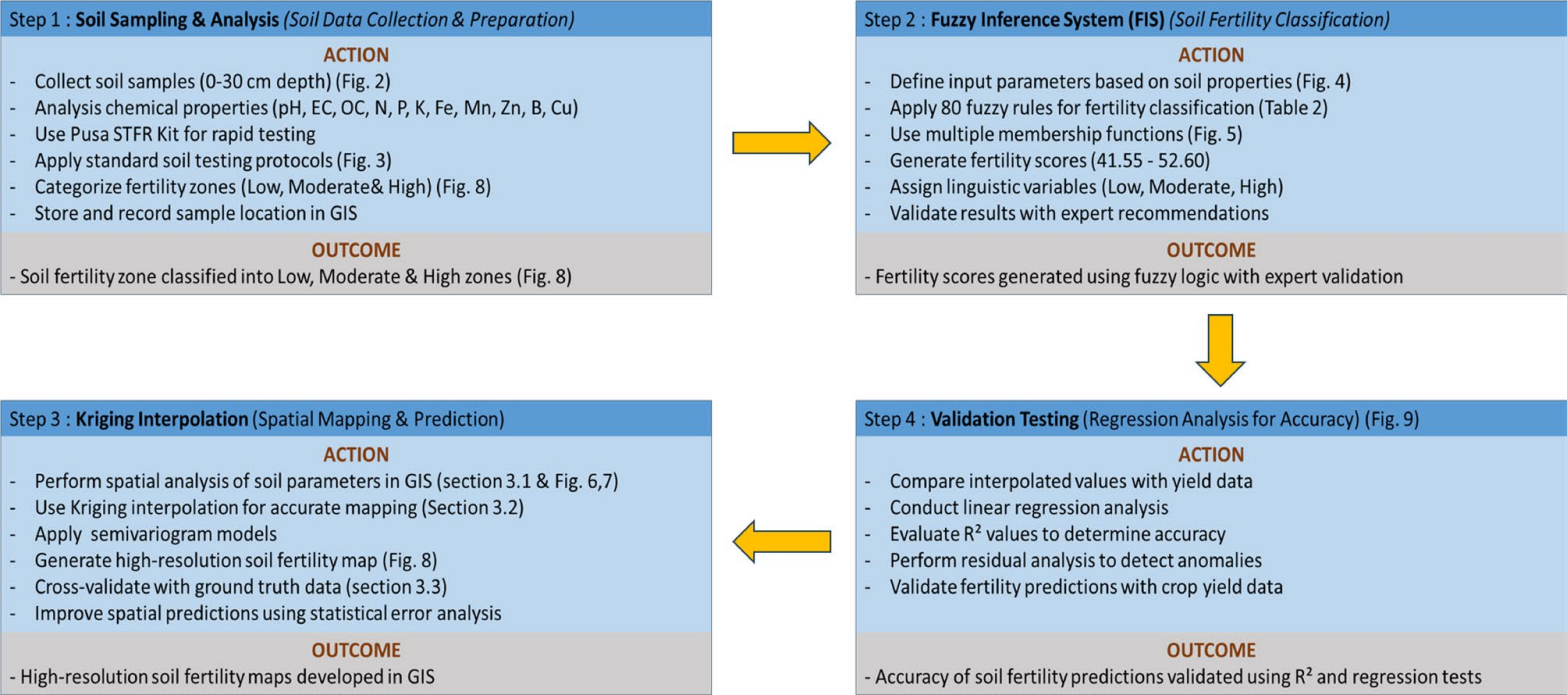
Decision Framework for Soil Fertility Assessment Using Fuzzy Inference System.

The study develops a composite model by integrating fuzzy inference systems (FIS) with geoinformatics (kriging technique) coupled with experimentation procedures. The modeling procedure is illustrated in Fig. 1, while Fig. 2 presents the Decision Framework for Soil Fertility Assessment Using Fuzzy Inference System.

FIS differs from other fuzzy systems in structure, rule formulation, and membership functions^29^. It excels in managing ambiguity and imprecision, offering robust solutions for resource allocation and prioritization in uncertain environments through precise rule-based logic^30^. When combined with expert systems, FIS provides a powerful framework for integrating expert knowledge and data to model complex agronomic and environmental processes^31^. It employs ‘IF–THEN’ rules with logical operators ‘OR’ and ‘AND’ to generate fuzzy scores corresponding to recommended outputs^10^.

For soil fertility assessment, the study classifies soil samples into three levels: high, medium, and low, based on chemical properties (pH, EC, OC), macronutrients (N, P, K), and micronutrients (Fe, Mn, Zn, Cu, B). Prior research confirms a strong correlation between soil fertility and crop productivity^32^. High fertility soils exhibit balanced pH, sufficient macronutrients, and optimal micronutrient levels, supporting crop growth. Medium fertility soils require targeted interventions to improve nutrient availability. Low fertility soils show severe macronutrient and micronutrient deficiencies, with low organic carbon content, making them less suitable for agriculture without significant amendments. Soil fertility determinants were categorized into chemical, physical, and biological factors.

To ensure spatially explicit fertility zoning, FIS was integrated with kriging interpolation, effectively addressing gaps in unsampled regions by predicting soil fertility values based on spatial correlations. Kriging, a geostatistical interpolation method, estimates unknown values by computing a weighted average of nearby measured points, considering both distance and spatial arrangement^33^. This technique not only generates estimates for unsampled areas but also quantifies prediction precision, making it a valuable tool in spatial analysis. The integration of FIS, Geographic Information Systems (GIS), and kriging enhances soil fertility assessment by combining expert knowledge, spatial data management, and statistical interpolation. FIS classifies soil fertility, GIS visualizes spatial data, and kriging predicts soil properties at unsampled locations, resulting in accurate soil fertility maps for precision agriculture^34,35^.

The model was extensively validated using real-world soil data from the Sabi River basin, demonstrating its adaptability to different agroecological zones. This approach enhances the accuracy of soil fertility zoning, enabling precise and informed decision-making for sustainable agricultural practices.

### Soil sampling and experimental analysis

**Fig. 3:**
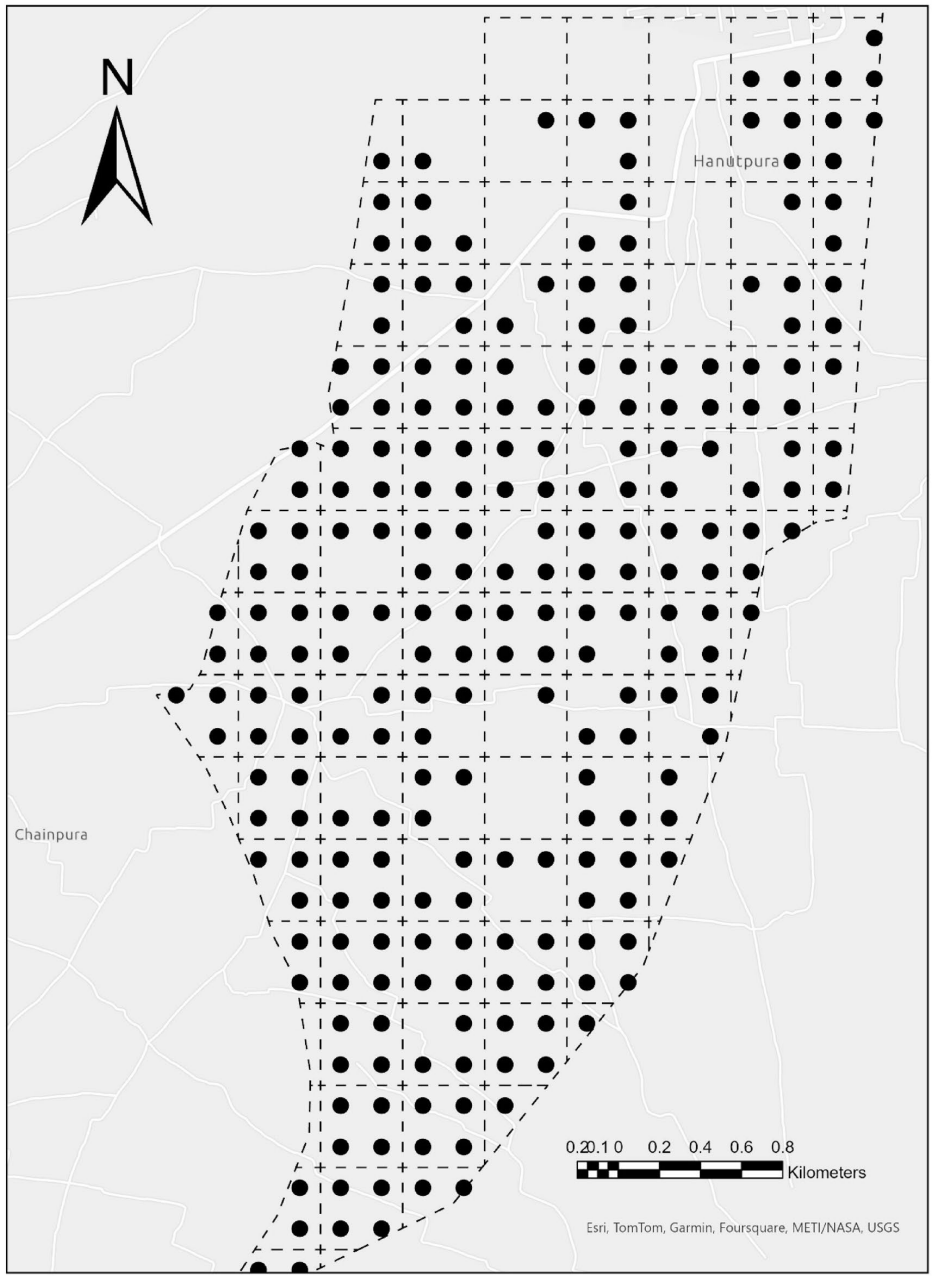
Locations of soil sampling in the study area using Fishnet Grid in ArcGIS Pro.

Prior to fertility evaluation, soil samples were collected from the top 0–30 cm soil depth across a 250-hectare study area, with one sample per hectare, ensuring comprehensive spatial coverage. The sampling locations were systematically selected using the Fishnet Grid tool in ArcGIS Pro, facilitating an even distribution of sample points shown in Fig. 3. This layer is critical for plant nutrient uptake and is strongly influenced by management practices and environmental conditions. The soil samples were air-dried, sieved through a 2 mm mesh, and analysed using the Pusa STFR (Soil Testing and Fertilizer Recommendation) Meter Kit. This kit is specifically designed for rapid and efficient determination of soil fertility parameters^36^.

**Fig. 4:**
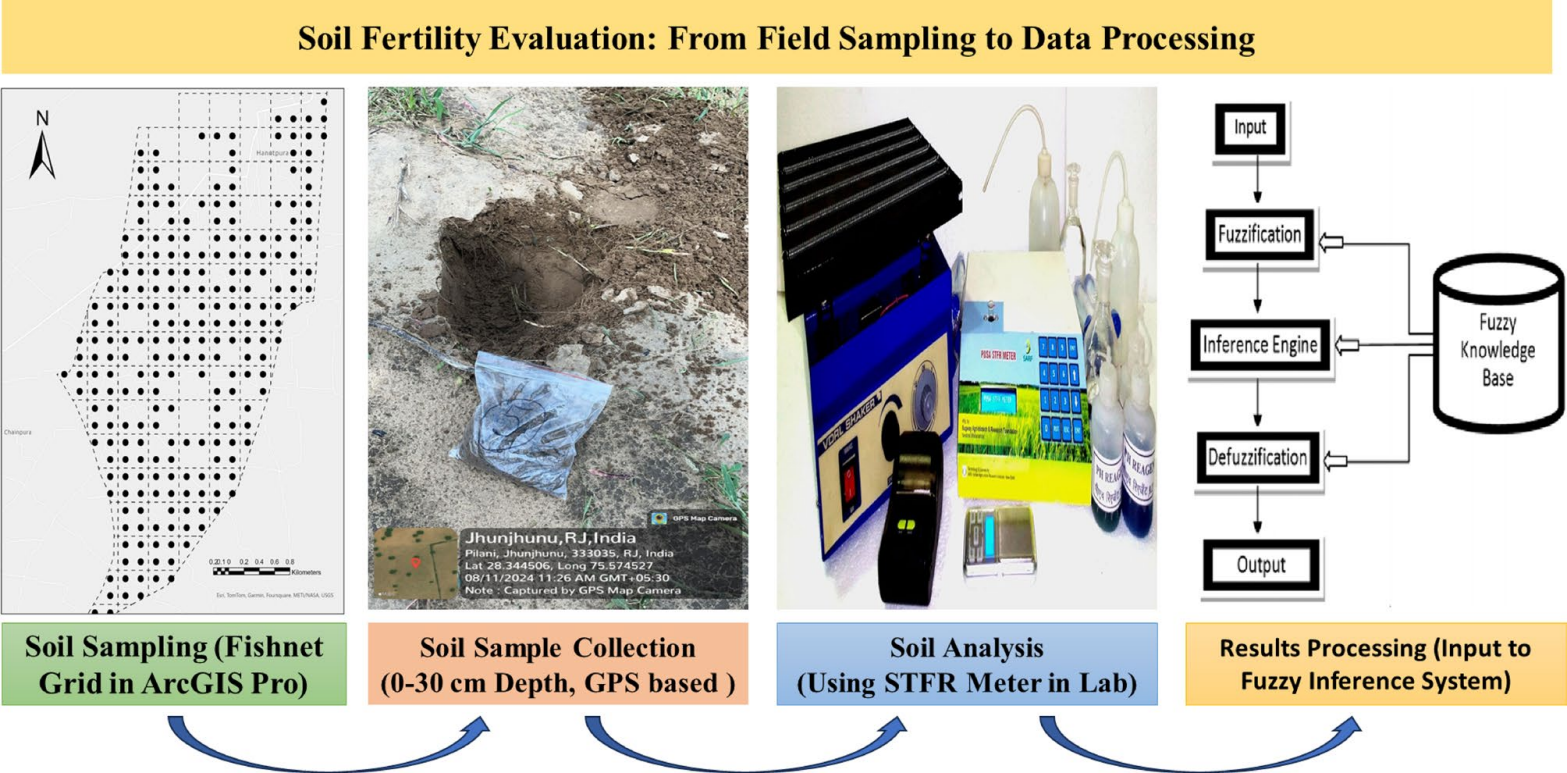
Soil fertility evaluation: From field sampling to data processing.

The Pusa STFR Meter Kit was employed to analyse essential soil properties, including pH, electrical conductivity (EC), organic carbon (OC), macronutrients (N, P, K), and micronutrients (Fe, Mn, Zn, B, Cu). The pH and EC were measured using electrodes into the kit, while OC was quantified based on a colorimetric method. Available nitrogen (N) was determined using alkaline permanganate extraction, phosphorus (P) using Olsen’s method for alkaline soils and Bray’s method for acidic soils, and potassium (K) using flame photometry. Micronutrients (Fe, Mn, Zn, Cu, B) were analysed through colorimetry in the Pusa STFR Kit, following standardized protocols^36^. The PUSA STFR Meter Kit, developed by ICAR-IARI, has been validated through peer-reviewed studies showing good correlation with standard laboratory methods for both macro- and micronutrients^37–39^, supporting its suitability for soil fertility research and nutrient management recommendations. Finally, the extracted nutrient concentration is converted to the standard unit kg/ha (Macronutrients), mg/kg (micronutrients) so that it may be correlated with soil fertilizer requirements. A The experimental investigation values were assessed using the Fuzzy Inference System (FIS) to classify the study area into high, medium, and low fertility zones, ensuring precise soil fertility evaluation. This classification, as illustrated in Fig. 4, was based on analysed soil parameters and rule-based inference, enabling a systematic approach to fertility zoning.

### Fuzzy inference system for processing experimental data

**Fig. 5:**
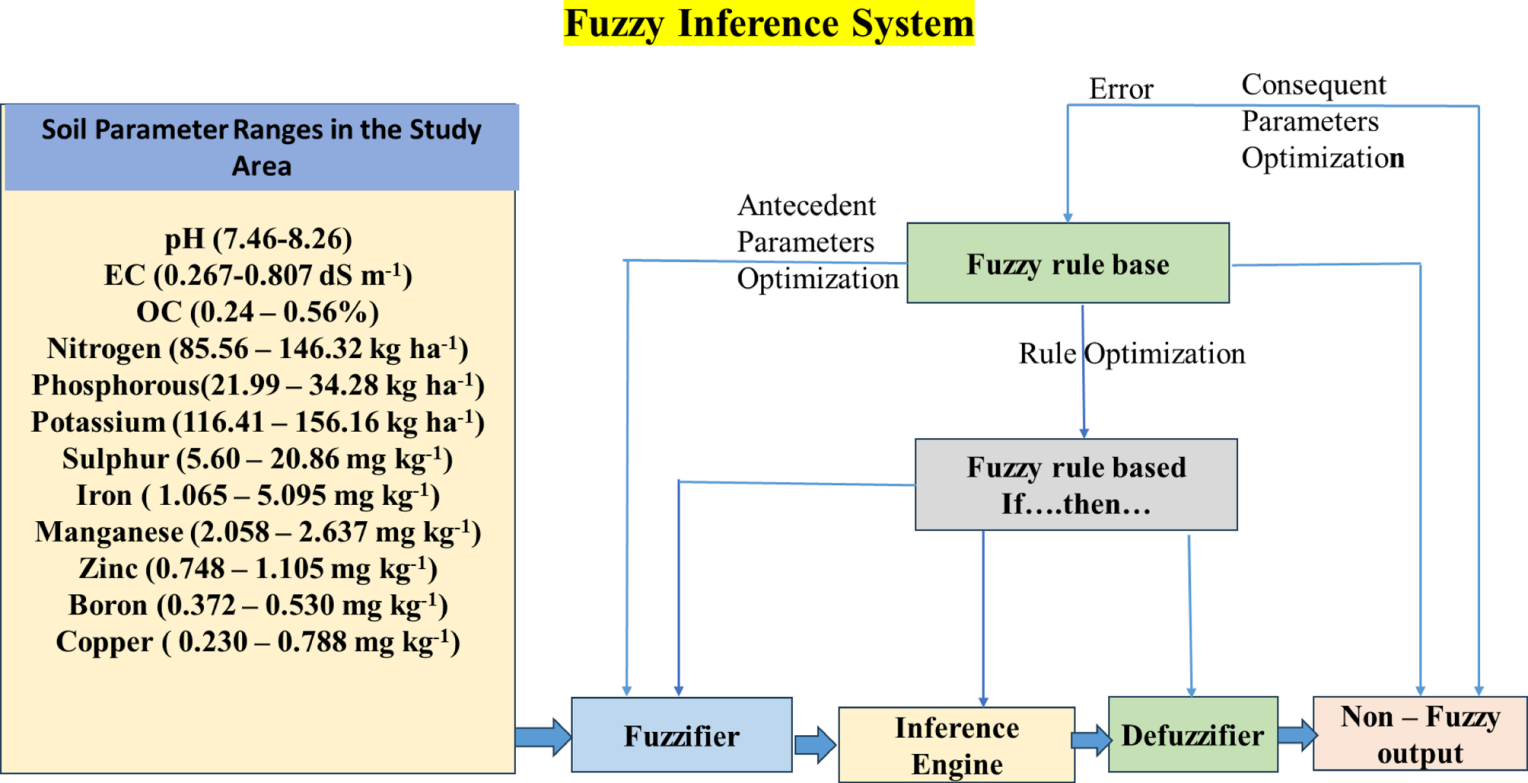
Schematic procedure of FIS adopted in the study to assess soil fertility.

The critical soil parameters were analysed using a MATLAB-based fuzzy inference system (FIS) to evaluate and classify soil fertility zones. For brevity reasons, brief procedure of FIS to determine the soil fertility levels is provided below and illustrated in Fig. 5. Further details can be obtained from Srinivas et al.^10^.

**Fig. 6:**
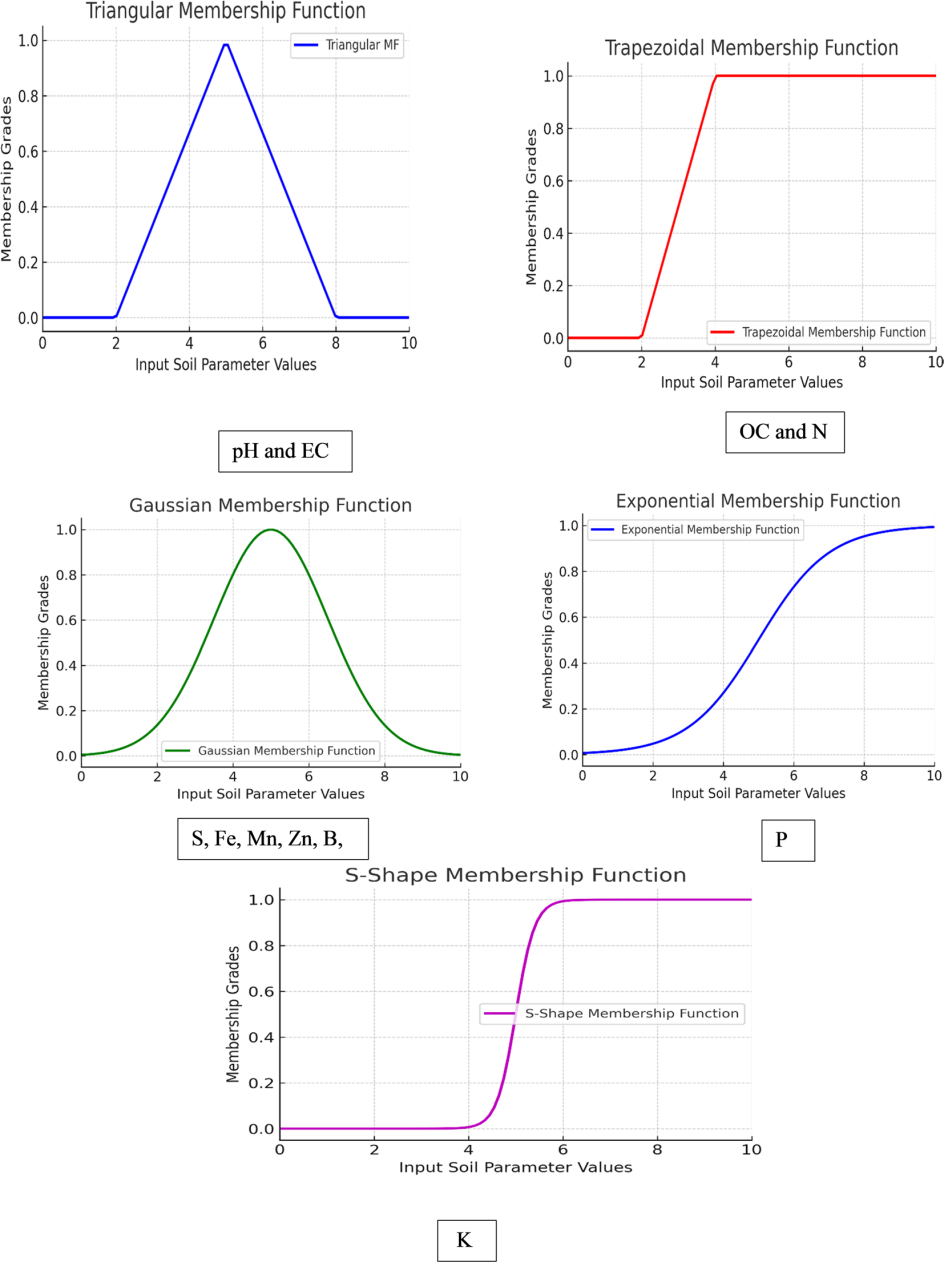
Membership functions used in FIS for soil fertility assessment.

- *Fuzzification process for soil fertility evaluation* The fuzzification process translated soil analysis (experimental investigation) results in linguistic terms, enabling the fuzzy inference system (FIS) to model uncertainty and variability in soil fertility parameters. Soil properties, including pH, EC, OC, N, P, K, Fe, Mn, Zn, B, and Cu, were classified into categories such as 'Low (L),' 'Medium (M),' and 'High (H)' based on expert recommendations and established guidelines^32,40^ . Various membership function (MF) shapes were applied: triangular MFs for parameters with linear transitions (e.g., pH, EC), trapezoidal MFs for broader ranges (e.g., OC, N), and Gaussian MFs for micronutrients reflecting natural variability. Input parameters were paired with the output variable, 'Soil Fertility,' categorized into zones like 'Low (L),' 'Medium (M),' and 'High (H).' The fuzzy inference system (FIS) was implemented using MATLAB (version R2022a, MathWorks Inc.) with the Fuzzy Logic Toolbox. The FIS process involved the following steps:
  (i) Input fuzzification—Twelve soil parameters (pH, EC, OC, N, P, K, S, Fe, Mn, Zn, B, and Cu) were fuzzified using triangular, trapezoidal, and Gaussian membership functions based on agronomic thresholds and expert domain knowledge. (ii) Rule base development—A total of 80 Mamdani-type fuzzy rules were formulated to relate various combinations of the input variables to fertility classes (e.g., low, medium, high). (iii) Fuzzy inference—The Mamdani inference mechanism was applied to generate fuzzy output values representing soil fertility scores. (iv) Defuzzification—The output fuzzy sets were converted into crisp fertility scores using the centroid method. These defuzzified values (ranging from 0 to 100) were then exported to ArcGIS Pro for spatial analysis. Ordinary kriging was employed to interpolate the fertility values and generate a continuous fertility surface across the study area. A spherical semivariogram model was used with a nugget of 5.2, sill of 42.8, and a range of 3500 meters, effectively capturing spatial autocorrelation and smoothing local variations. This integrated fuzzy logic–geostatistical framework enabled the robust delineation of soil fertility zones, offering a scientifically grounded and spatially coherent approach^41,42^. This approach effectively captured soil variability, ensuring robust fertility zoning to support precision agriculture^10,43^.
- *Membership functions* Membership functions (MFs) are critical components in the fuzzy inference system (FIS), representing the degree of participation of each input parameter in a given fuzzy set. In this study, membership functions were used to model the uncertainty and variability of soil fertility parameters, including pH, EC, OC, N, P, K, Fe, Mn, Zn, B, and Cu. The MFs were selected and defined based on expert recommendations and established soil fertility standards, ensuring the accurate representation of soil characteristics. All MFs were designed to satisfy the standard constraints of fuzzy sets: a range between 0 and 1 and uniqueness for each $x \in X,\mu_{A } \left( x \right)$ must be sole.
  The selected membership functions include various shapes to best fit the characteristics of the soil fertility parameters:

*Triangular membership function*. Triangular MFs were used for parameters with clear linear transitions, such as pH and EC (Fig. 6). This MF provides simplicity and computational efficiency^10^. The function is defined using Eq. (1):

1 $$\mu_{A} \left( x \right) = \left\{ {\begin{array}{*{20}c} 0 & {x \le \alpha_{min} } \\ {\frac{{x - \propto_{min} }}{{\beta - \alpha_{min} }}} & {x \epsilon \left( {\alpha_{min} ,\beta } \right)} \\ {\frac{{\alpha_{max} - x}}{{\alpha_{max} - \beta }}} & {x \epsilon \left( {\beta ,\alpha_{max} } \right)} \\ 0 & { x \le \alpha_{max} } \\ \end{array} } \right.$$

*Trapezoidal membership function*: For parameters with broader ranges, such as organic carbon (OC) and nitrogen (N), trapezoidal MFs were used to account for gradual transitions (Fig. 6). This MF is effective in modeling ranges with flat-topped regions^32^. The function is defined using Eq. (2):

2 $$\mu_{A} \left( x \right) = \left\{ {\begin{array}{*{20}c} 0 & {if x \le \alpha_{min} } \\ {\frac{{x - \alpha_{min} }}{{\beta - \alpha_{min} }}} & {x \epsilon \left( {\alpha_{min} ,\beta_{1} } \right)} \\ 1 & {x \epsilon \left( {\beta_{2} ,\alpha_{max} } \right)} \\ 0 & {x \ge \alpha_{max} } \\ \end{array} } \right.$$

Gaussian membership function: Micronutrients (S, Fe, Mn, Zn, B, Cu) were modelled using Gaussian MFs due to their natural variability and distribution. The function is expressed using equation (3):

3 $$\mu_{A} \left( x \right) = e^{{ - \alpha \left( {x - \beta } \right)^{2} }}$$

Exponential-like membership function: For phosphorous (P), which requires rapid decay modeling, an exponential-like MF was applied to capture its variability effectively. The MF is expressed using Eq. (4):

4 $$\mu_{A} \left( x \right) = {\raise0.7ex\hbox{$1$} \!\mathord{\left/ {\vphantom {1 {\left( {1 + \gamma \left( {x - \beta } \right)} \right.}}}\right.\kern-0pt} \!\lower0.7ex\hbox{${\left( {1 + \gamma \left( {x - \beta } \right)} \right.}$}}2$$

S-Shape membership function (MF): This MF allows for smooth transitions in data representation in data representation^43^. It was used for parameters like potassium (K) using Eq. (5):

5 $$\mu_{A} \left( x \right) = \left\{ {\begin{array}{*{20}c} 0 & {x \le \alpha_{min} } \\ {2\left( {\frac{{x - \propto_{min} }}{{\beta - \alpha_{min} }}} \right)^{2} } & {x \epsilon \left( {\alpha_{min} ,\beta } \right)} \\ {1 - 2\left( {\frac{{x - \propto_{min} }}{{\beta - \alpha_{min} }}} \right)^{2} } & {x \epsilon \left( {\beta ,\alpha_{max} } \right)} \\ 0 & {x \le \alpha_{max} } \\ \end{array} } \right.$$

The selection of MFs was guided by expert input and domain-specific literature to ensure that the fuzzy sets accurately captured the characteristics of the soil fertility parameters, enabling precise and meaningful classification of soil fertility zones.

General Terms Across All Formulae:

- $\mu_{A} \left( x \right) :$ Membership value of input *x* in fuzzy set A. Represents the degree of belonging of *x* to the fuzzy set A.
- ***x***: Input variable (e.g., soil pH, EC, OC, N, P, K,S, Fe, Mn, Zn, B, Cu).
- $\alpha_{min}$: Lower bound of the membership function (minimum value of the range).
- $\alpha_{max}$: Upper bound of the membership function (maximum value of the range).
- *β*: Parameter defining the peak or center of the membership function (where membership = 1 or 0.5, depending on the MF type).
- *γ*: Decay or spread parameter (controls the shape of the membership function).

*Developing expert fuzzy inference rules*: The relationships among soil fertility parameters and the resulting fertility zones were used to develop expert fuzzy inference rules, integrating input parameters such as pH, EC, OC, N, P, K, Fe, Mn, Zn, B, and Cu. These rules, formulated as IF–THEN statements and linked using fuzzy operators (AND or OR), were established through extensive consultation with expert panel to ensure practical relevance shown in Table 1. The experts, comprising soil scientists, fertility specialists, and geospatial analysts, contributed their expertise in soil nutrients, remote sensing, and precision farming, ensuring scientifically valid soil fertility classification rules. Mathematically, the total possible rules are defined as R = [number of linguistic variables]^(number of criteria)^, which, for three linguistic classes (Low, Medium, High) and twelve input parameters, results in 531,441 potential combinations. Recognizing the impracticality of validating all combinations, 80 scientifically sound rules were formulated to reflect critical soil fertility interactions and observed data patterns. These rules (Table 1) capture the complex relationships among soil parameters and enable the fuzzy inference system to generate reliable soil fertility zones, ensuring actionable insights for effective soil management.

**Table 1:** An illustration of fuzzy inference rules formed using input parameters (P1, P2, and P3), membership functions (MF) operators for soil fertility classification.

S.No	P1	MF 1	Operator	P2	MF 2	Operator	P3	MF 3	Soil fertility
1	pH	Low	AND	OC	Low	AND	N	Low	Poor
2	pH	Medium	AND	P	Low	AND	K	Low	Poor
3	pH	High	AND	OC	Medium	AND	N	Medium	Moderate
4	EC	High	AND	Zn	Low	AND	Mn	Low	Poor
5	OC	High	AND	Fe	High	AND	Cu	Medium	High
6	N	Low	AND	P	Medium	AND	K	High	Moderate
7	B	Low	AND	Cu	Low	AND	Mn	Low	Poor
8	P	High	AND	K	High	AND	Zn	High	High
9	EC	Medium	AND	Fe	Medium	AND	B	Low	Moderate
10	pH	Medium	AND	OC	High	AND	N	High	High

Fuzzy Inference System Rules derived by experts to assess soil fertility *Integration of Rule Outputs and Defuzzification*: FIS executed the defined rules to evaluate soil fertility zones, aggregating the outputs into a single membership function that represented the overall soil fertility status^44,45^. This aggregated membership function was then defuzzified using the centroid method, to derive crisp scores. These crisp values categorized soil into distinct fertility zones—Low, Medium, and High—based on the analysed soil parameters^46^. The highest crisp score for each location indicated the recommended soil fertility zone, enabling targeted agricultural interventions. This approach provided a robust methodology for translating fuzzy soil data into actionable insights for precision farming and sustainable soil management.

### Geoinformatics: Kriging interpolation to estimate soil fertility

Kriging is a geostatistical interpolation technique which consider both the distance and degree of variation was employed to estimate soil fertility values for unsampled areas, enabling the development of continuous soil fertility zoning maps using ArcGIS Pro^47^. Ordinary Kriging, which assumes a constant mean within local neighbourhoods, was applied to interpolate soil parameters such as pH, EC, OC, N, P, K, Fe, Mn, Zn, B, and Cu across the entire study area. The spatial random function $Z\left( s \right)$ was expressed as $Z\left( s \right) = \mu + \in \left( s \right)$ where $\mu$ represents the constant means and $\in \left( s \right)$ accounts for variations around the mean. The semivariogram $\gamma \left( h \right) - sill - Cov\left( h \right)$ was used to model spatial variability, with terms including the range (distance where correlation diminishes), sill (maximum variance), and nugget (variability at zero distance). Gaussian, spherical, and exponential semivariogram models were evaluated to fit the soil data, with the spherical model preferred for its smooth transitions. The selection of an appropriate semivariogram model is crucial for accurately capturing spatial dependencies in soil properties. To compare different interpolation methods, the mean error (ME) and root mean square error (RMSE) were calculated from the measured and interpolated values at each sample location using Eqs. 6 and 7. These statistical metrics help assess the accuracy and efficiency of spatial interpolation techniques in predicting soil fertility parameters across the study area:

6 $${\text{ME }} = \frac{1}{n}\mathop \sum \limits_{i = 1}^{n} \left\{ {z\left( {x_{i} } \right) - z^{*} \left( {x_{i} } \right)} \right\}$$

7 $${\text{RMSE }} = \sqrt {\frac{1}{n}\mathop \sum \limits_{i = 1}^{n} \left\{ {z\left( {x_{i} } \right) - z^{*} \left( {x_{i} } \right)} \right\}^{2} }$$

where $z\left( {x_{i} } \right)$ is the observed value at location i, $z^{*} \left( {x_{i} } \right)$ is the interpolate value at location I,and n is the sample size. Ideally ME should tend to zero and RMSE should be small as possible to indicate less error and more accurate spatial interpolator, respectively. Based on these criteria, the spherical model yielded the best values, making it the most suitable choice for spatial interpolation in this research. Kriging complemented the FIS by estimating values in unsampled areas, filling spatial gaps to create detailed fertility maps. Together, these methods provided a robust framework for evaluating soil fertility and supporting precision agriculture and soil management strategies.

### Yield sampling

A systematic grid sampling strategy was employed to collect yield data from the study area. The area was divided into a grid of 200 m × 200 m cells, with one sampling point selected at the center of each cell. Yield data was collected for four major crops [pearl millet], [wheat], [mustard], and [barley] over multiple growing seasons. At each sampling point, a quadrat of [size, e.g., 1 m^2^] was used to harvest the crop, and the yield was measured using a digital scale. If a selected crop was unavailable at a sampling point during a particular season, yield data from the previous season was used as a substitute. The geographical coordinates of each sampling point were recorded using a GPS device. To improve accuracy, the yield data from multiple seasons were averaged using the Eq. 8.

8 $$Y = \frac{1}{N}\mathop \sum \limits_{i = 1}^{n} Y_{i}$$

where Y is the yield in the ith season, and n is the total number of seasons. The sampled yield values were validated against actual harvested yield data obtained through farmer surveys. The accuracy of the sampled yield data was assessed using the Root Mean Square Error (RMSE), which quantifies the difference between the sampled yield (Y sample) and the actual harvested yield (Y harvested). The RMSE was calculated using Eq. 9:

9 $${\text{RMSE }} = \sqrt {\frac{1}{n}\mathop \sum \limits_{i = 1}^{N} \left( {Ysample - Y harvested} \right)^{2} }$$

where N is the total number of sampling points. The final dataset was pre-processed to remove outliers using the Interquartile Range (IQR) method, ensuring its reliability for further analysis.

Crop yield data was systematically collected during the respective harvest seasons for pearl millet, wheat, mustard, and barley over multiple growing seasons within the defined study period. The study area was divided into a uniform grid of 200 m × 200 m, and at the center of each grid cell, yield sampling was conducted. Crop yield data was collected primarily from these same grid locations as the soil samples. If the target crop was not present at a grid point in a particular season, yield data for the previous crop at that location was recorded, assisted by farmer inputs. At each sampling point, crop yield was measured using a 1 m^2^ quadrat, and harvested biomass was weighed with a digital scale. The yield obtained from 1 m^2^ was converted to kg/ha using the formula: kg ha^−1^ = (yield in kg from 1 m^2^) × 10,000. The geographical coordinates of each sampling point were recorded using GPS to ensure spatial accuracy. Yield data were collected consistently across the study area to maintain alignment with the soil sampling grid and were validated through farmer-reported harvested yields. While soil sampling follows standardized protocols for consistent and repeatable data collection, crop yield sampling lacks a universally coordinated scheme because yield is influenced by dynamic factors such as weather variability, pest and disease pressures, irrigation, and management practices. This complexity makes it difficult to establish a single, uniform yield sampling protocol. The proposed approach addressed this by aligning yield sampling points with the soil grid to enhance spatial–temporal relevance.

### Study area

**Fig. 7:**
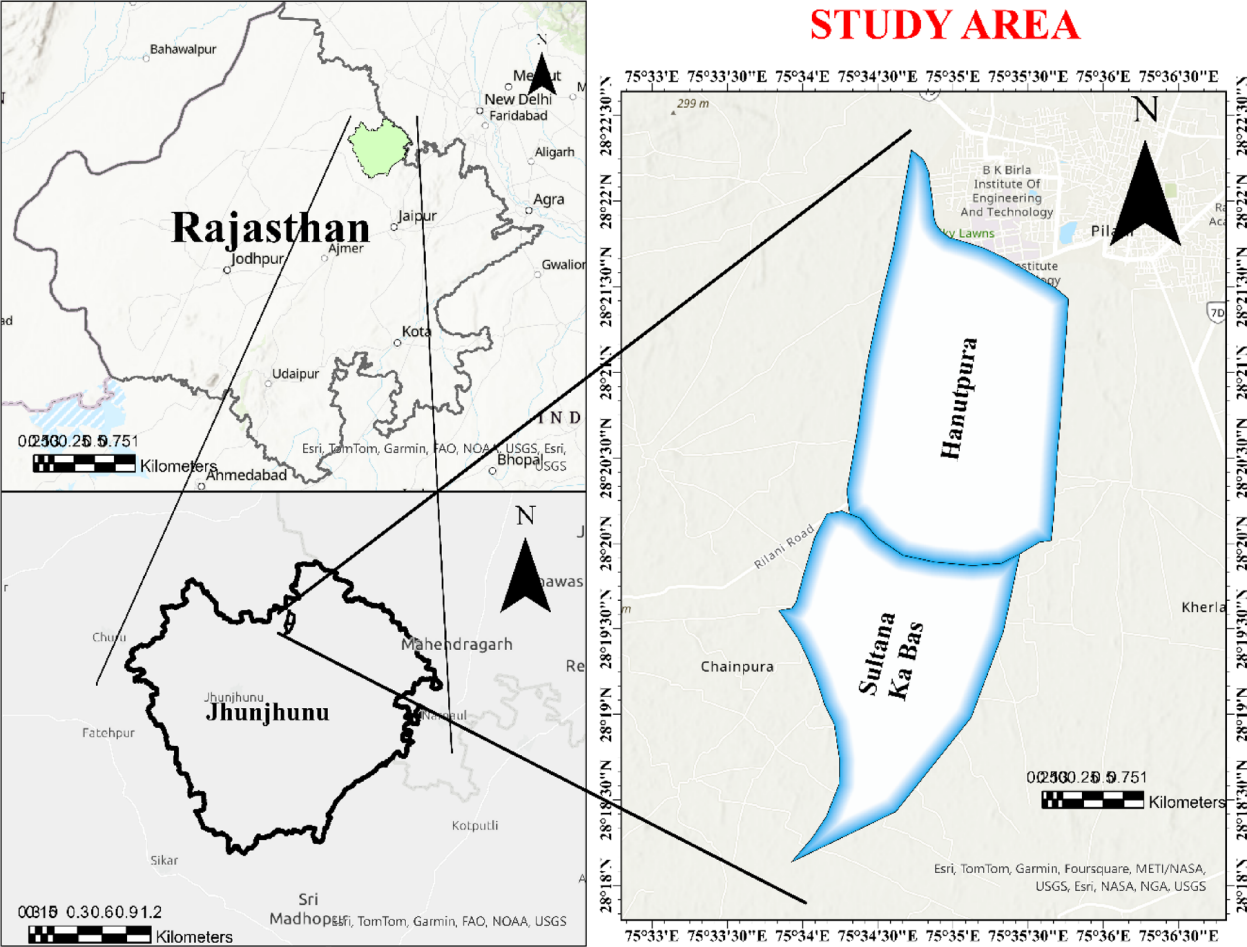
Study area representing the two villages for soil fertility assessment.

The study was demonstrated in Hanutpura and Sultan Ka Bas villages of Jhunjhunu district, Rajasthan, India (Fig. 7), situated within the geographic coordinates with its north latitude from 28° 24′ 44.00″ to 28° 16′ 29.28″ and east longitudes 75° 37′ 49.89″ to 75° 30′ 46.89″. The region falls under the agro-ecological sub region as per ICAR. The area receives an average annual rainfall of 444.5 mm, primarily during the monsoon season, and experiences semi-arid climatic conditions with high temperature variations. Agriculture is a major livelihood activity, with pearl millet (bajra), wheat, mustard, and barley being the predominant crops. The region’s soil is characterized by low to moderate fertility, influenced by sandy loam texture and limited organic matter content. Unsustainable agricultural practices, including intensive cropping and inadequate nutrient management, have led to soil degradation and declining productivity. Given these challenges, the study area was selected to evaluate soil fertility variability and propose scientifically informed soil management strategies for sustainable agricultural practices.

The study systematically assessed soil fertility variability in Hanutpura and Sultan Ka Bas villages using a Fuzzy Inference System (FIS) integrated with GIS-based kriging interpolation. A structured expert-driven approach was adopted, where eight soil scientists, agronomists, and geospatial experts contributed insights to develop a rule-based fuzzy classification system for soil fertility zoning. A total of 80 fuzzy rules were formulated to define the relationships between soil chemical properties (pH, EC, OC, N, P, K, S, Fe, Mn, Zn, B, Cu) and fertility categories (unproductive, low, medium, and productive). Soil samples were collected from a 0–30 cm depth following a fishnet grid approach in ArcGIS Pro, ensuring spatially representative data distribution. The experts assigned triangular, trapezoidal, gaussian, exponential, and S-shaped membership functions to classify soil fertility variations. The fuzzy rule base was implemented in MATLAB, and the spatial distribution of fertility was interpolated using a spherical semivariogram model in ArcGIS Pro, producing a soil fertility map that identified very low, low, and moderate fertility zones. The developed fuzzy rule base (Table 2) and its corresponding rule-based matrix provide an in-depth understanding of soil fertility dynamics, ensuring scientific recommendations for targeted soil management and sustainable precision agriculture practices.

**Table 2:** Fuzzy rule base for soil fertility classification.

Description	Antecedent	Consequent	Weight	Connection
pH = Excessive-Acid, output = Unproductive	[1,0,0,0,0,0,0,0,0,0]	1	1	1
EC = Strongly-Saline, output = Unproductive	[0,0,4,0,0,0,0,0,0,0]	1	1	1
OM = Low & N = Low, output = Unproductive	[0,1,0,1,0,0,0,0,0,0]	1	1	1
Zn = Low & B = Low, output = Unproductive	[0,0,0,0,0,0,0,0,1,1]	1	1	1
pH = Acid, output = Low	[2,0,0,0,0,0,0,0,0,0]	2	1	1
OM = Low, output = Low	[0,1,0,0,0,0,0,0,0,0]	2	1	1
EC = Slightly-Saline & N = Low, output = Low	[0,0,2,1,0,0,0,0,0,0]	2	1	1
P = Low &K = Sufficient, output = Low	[0,0,0,0,1,2,0,0,0,0]	2	1	1
pH = Neutral, output = Medium	[3,0,0,0,0,0,0,0,0,0]	3	1	1
OM = Medium, output = Medium	[0,2,0,0,0,0,0,0,0,0]	3	1	1
EC = Non-saline & N = Sufficient, output1 = Medium	[0,0,1,2,0,0,0,0,0,0]	3	1	1
pH = Neutral & OM = Medium & EC = non-Saline, output = Medium	[3,2,1,0,0,0,0,0,0,0]	3	1	1
pH = Neutral & OM = High & N = High,output = Productive	[3,3,0,3,0,0,0,0,0,0]	4	1	1
EC = Non-Saline & P = High & K = High, output1 = Productive	[0,0,1,0,3,3,0,0,0,0]	4	1	1
OM = Very-high & EC = Non-Saline & N = High & K = High, output = Productive	[0,4,1,3,0,3,0,0,0,0]	4	1	1
Fe = Medium & Zn = High & B = High, output = Productive	[0,0,0,0,0,0,2,0,3,3]	4	1	1

## Results and discussion

### Spatial variation of soil fertility parameters

The spatial variability in the study area was obtained through a combination of on-ground soil sampling followed by experimentation and GIS-based spatial analysis. Soil samples were collected systematically using the fishnet grid technique in ArcGIS Pro and analysed for key fertility parameters including organic carbon (OC), electrical conductivity (EC), pH, total nitrogen (N), available phosphorus (P), available potassium (K), Sulphur (S), iron (Fe), manganese (Mn), zinc (Zn), boron (B), and copper (Cu) were analysed, focusing on the upper 30 cm of the surface soil using the PUSA STFR Meter. The obtained data were then digitized and interpolated using the kriging method in GIS, enabling spatial prediction of soil fertility across unsampled locations. The descriptive statistics of the measured soil properties are presented in Table 2, highlighting their central tendency and variability, which informed the fuzzy inference process for fertility classification^48^. Although the fertility zones are model derived outputs, statistical comparisons such as ANOVA or Welch’s ANOVA can be useful for assessing inter-zone variability in underlying soil properties. Readers are encouraged to explore such analyses where statistical assumptions are met, and future studies will incorporate this approach to further validate zonation outcomes.

**Fig. 8:**
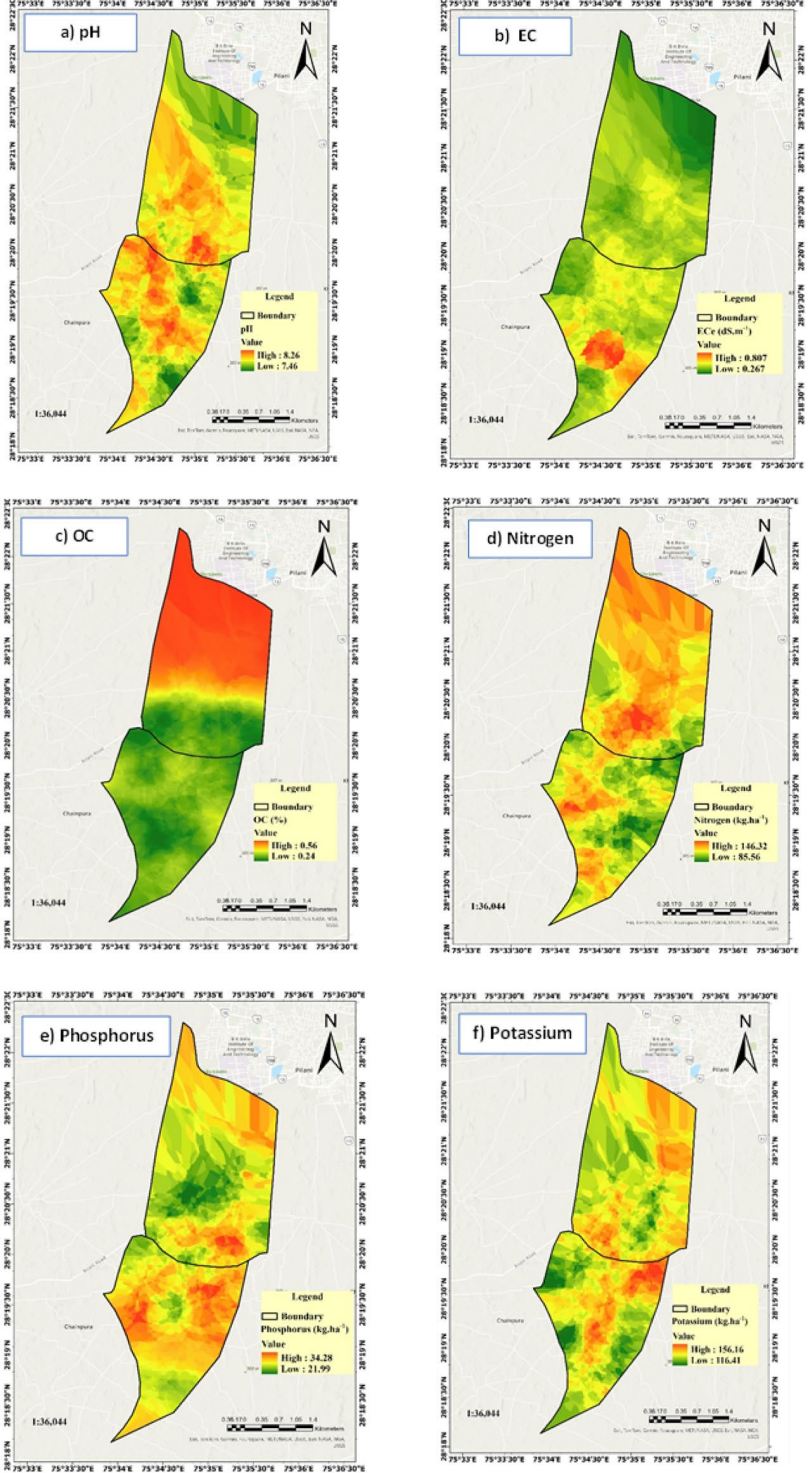

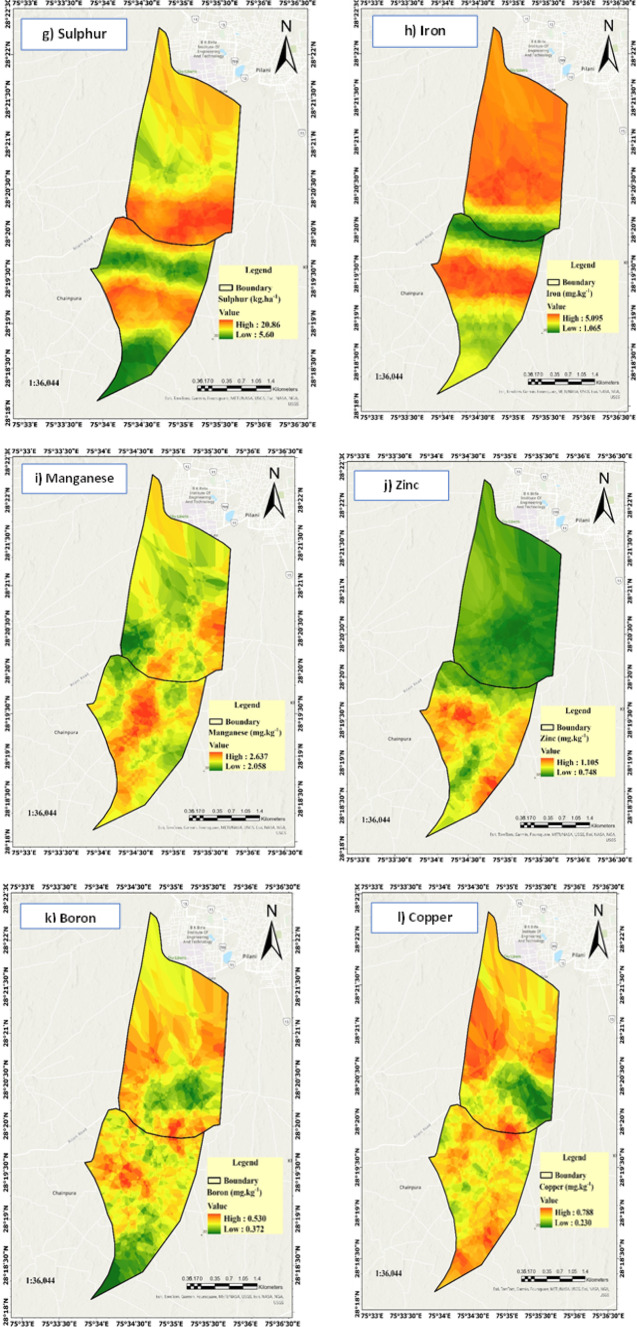
(**a**–**f**) Zonation of soil chemical parameters (0–30 cm) including pH, ECe, OC, N, P and K in the study area. (**g**–**l**) Zonation of soil nutrient elements (0–30 cm) including S, Fe, Mn, Zn, B and Cu in the study area.

This approach effectively captured variability in soil properties and facilitated precise classification of fertility zones. Soil fertility parameters. The soil property maps (Fig. 8) were generated using the geostatistical kriging interpolation method in ArcGIS Pro. The process began with exploratory data analysis to check for normality, outliers, and trends in the soil parameter data. Semi-variogram analysis was then performed to characterize spatial autocorrelation and quantify spatial variability for each parameter. Different theoretical models (spherical, exponential, and Gaussian) were evaluated, and the spherical model provided the best fit for most parameters based on lowest residual sum of squares and highest coefficient of determination (R^2^)^49^. The variogram parameters—nugget, sill, and range—were optimized to reflect spatial continuity and structure of the data. Ordinary kriging was applied to predict soil property values at unsampled locations, with cross-validation used to assess model performance and minimize prediction error^50^. The maps were produced at a spatial resolution of 10 m, providing detailed zonation for precise soil fertility management.

The upper soil layer was selected due to its critical role in nutrient availability and plant root interactions, as most plant roots and microbial activity are concentrated in this layer^51,52^. Furthermore, the surface soil is more influenced by management practices, such as fertilization and tillage, making it the most dynamic zone for soil fertility assessment^53^ (Fig. 3). For proper fertilizer management in pearl millets (bajra) cultivation, it is essential to have comprehensive information about soil chemical properties and nutrient levels to enhance crop performance and ensure sustainable agricultural practices^52,54^. Accurate assessment of soil fertility parameters allows for precise input application, reducing wastage and improving yields.

Limited information on the variability of soil chemical properties in several semiarid regions of India, particularly regarding plant nutrients, often leads to the blanket application of fertilizers^55,56^. This practice results in an unequal distribution of inputs, reducing efficiency and sustainability (Keshavarzi et al. 2018). Some spots tend to receive more, while others receive less than required.

The soil in the study area exhibits a basic reaction, with pH ranging from 7.46 to 8.64, suitable for cultivation. The pH values ranged from 7.46 to 8.26, indicating variations in soil alkalinity across different locations (Fig. 8a). This spatial variability in soil reaction affects nutrient availability, particularly of micronutrients like zinc and iron, which are less available in alkaline soils^51^. Site-specific amendments, such as sulphur or gypsum, may be required to optimize nutrient uptake and enhance crop yield in high-pH zones^57^. EC ranged from 0.267 to 0.807 dS m⁻^1^, indicating non-saline to slightly saline conditions, which can influence nutrient availability and water uptake, potentially affecting crop growth and yield^58^ (Fig. 8b). The soil organic carbon content exhibited 0.24% to 0.56% range, which is relatively low and may affect soil fertility, water retention, and microbial activity^59^. Studies suggest that increasing OC improves nutrient availability and crop productivity, but the impact diminishes beyond a threshold concentration^52^ (Fig. 8c). The total nitrogen content ranged from 85.56 to 146.32 kg ha^−1^. The relationship between soil reaction and nitrogen availability reflects the influence of pH on nitrogen mineralization and nutrient retention^60^. Although nitrogen levels appear sufficient for crop cultivation, localized interventions, such as nitrogen fertilization in low-concentration zones, could improve productivity (Fig. 8d). Available phosphorus ranged from 21.99 to 34.28 kg ha^−1^ and available potassium from 116.41 to 156.16 kg ha^−1^, with both nutrients exhibiting variability across all regions (Fig. 8e,f). Low and high concentrations of phosphorus and potassium were distributed throughout the area, reflecting the heterogeneous nature of soil fertility. This uneven distribution emphasizes the importance of site-specific nutrient management to address deficiencies and enhance crop productivity. The sulphur content ranged from 5.60 to 20.86 mg kg^−1^. Sulphur plays a crucial role in protein synthesis, enzyme activation, and nitrogen metabolism in plants. Deficiencies can result in chlorosis, stunted growth, and reduced crop yield, particularly in sulphur-demanding crops like oilseeds and cereals^61^ (Fig. 8g).

The variability in micronutrient concentrations in the study area can be attributed to differences in soil pH, organic carbon content, and macronutrients. Fe levels, ranging from 1.065 to 5.095 mg kg^−1^ (Fig. 8h), are influenced by soil pH, as Fe becomes less available in alkaline conditions due to its precipitation as insoluble oxides^62^. Higher Fe concentrations may result from localized organic matter inputs, which enhance Fe availability through chelation. Mn levels, ranging from 2.058 to 2.637 mg kg^−1^, reflect its sensitivity to redox conditions and pH (Fig. 8i). Low Mn availability in some areas could result from oxidation in well-drained soils, whereas higher levels are often associated with poorly drained conditions that favor Mn solubility^63^. Zn concentrations, varying between 0.748 and 1.105 mg kg^−1^, are affected by pH, with Zn becoming unavailable in alkaline soils due to adsorption to clay particles and calcium carbonate^64^ (Fig. 8j). Variations in Zn levels may also result from differential application of fertilizers and organic amendments. Boron with concentrations between 0.372 and 0.530 mg kg^−1^, shows low availability across the area, likely due to leaching in sandy soils and reduced mobility in high pH conditions. Boron deficiency is a common issue in semi-arid regions with low organic matter^65^ (Fig. 8k). Cu levels, ranging from 0.230 to 0.788 mg kg^−1^ (Fig. 8l), are similarly influenced by soil pH and organic carbon content. Low Cu levels in some areas may result from its fixation in organic complexes or low parent material content. These findings highlight the need for site-specific soil management strategies, including micronutrient supplementation and pH correction, to address deficiencies and improve selected crops productivity.

### Soil fertility zonation using kriging

This research applied FIS to assess the fertility potential specifically for bajra (pearl millet), wheat, mustard and barley incorporating essential soil parameters to guide precise agricultural planning. The soil fertility factors, including chemical and nutrient elements, were fuzzified using multiple membership functions. In order to determine the degree of membership function for each factor, the range and adequacy values of parameters a and b were defined based on a yield for selected crops production. Using the FIS, different soil parameters were incorporated to evaluate soil fertility. FIS outputs were integrated into GIS by using kriging interpolation technique to create the final soil fertility zonation map for selected crops production. The spherical semivariogram model in Kriging interpolation was used in ArcGIS Pro to capture the spatial dependence of soil fertility parameters, effectively smoothing local variations and ensuring accurate fertility predictions across unsampled locations^66^. While ordinary kriging was applied to interpolate fuzzy soil fertility scores and account for spatial autocorrelation of these values, we recognize that regression kriging could further enhance zonation accuracy by incorporating auxiliary variables^67,68^. This will be explored in future work as we integrate additional spatial covariates such as remote sensing data and terrain attributes.

**Fig. 9:**
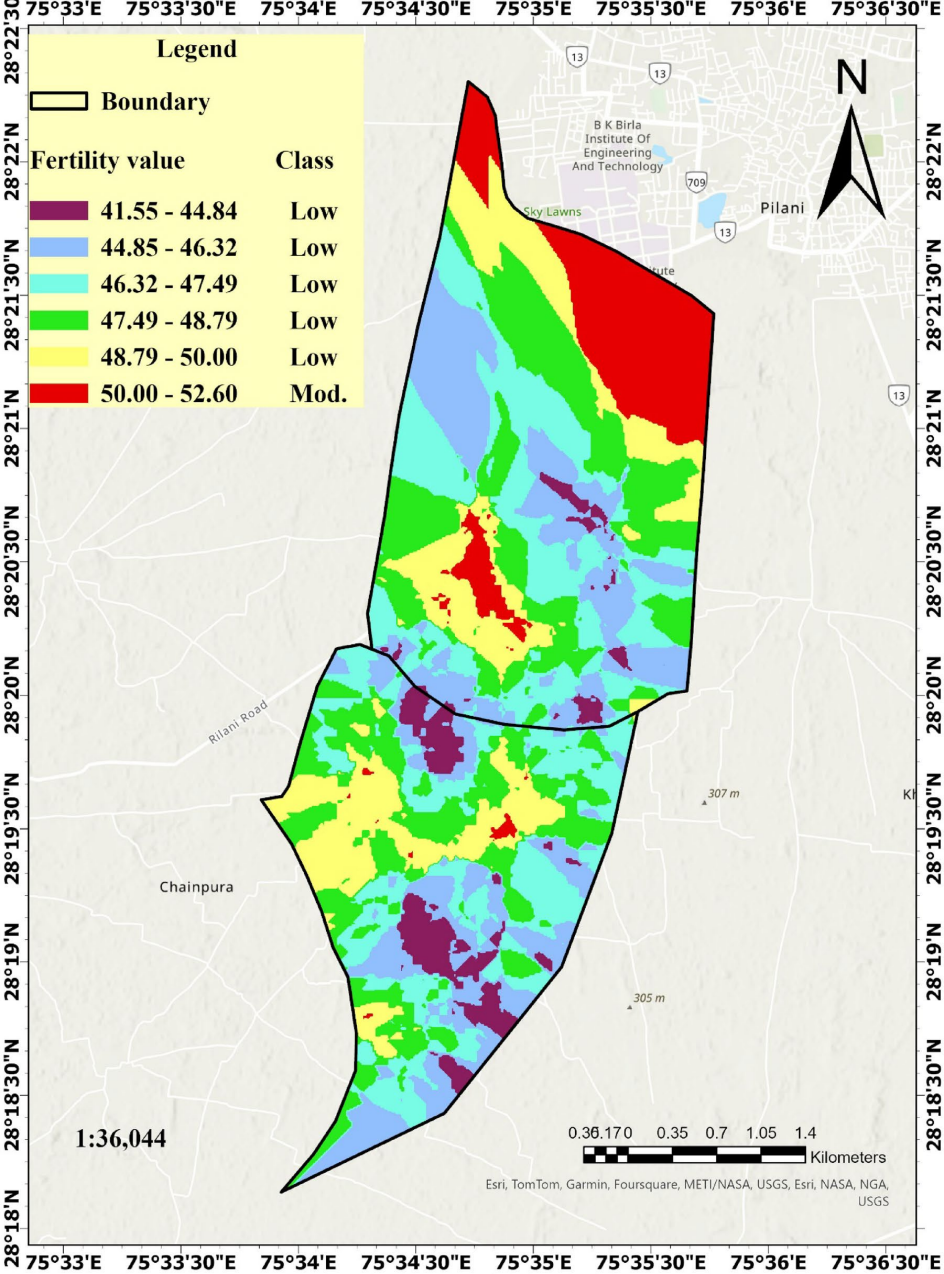
Zonation of fertility using fuzzy inference system and kriging interpolation technique.

The soil fertility values, analysed on a scale of 0–100, ranged from 17.4 to 52.6, which were classified as low to moderate fertility levels (Table 3, Fig. 9). Although the classification framework included five categories—very low, low, moderate, high, and very high (Table 4)—the soil fertility values obtained through the FIS and kriging fell only within the low and moderate ranges (17.4–52.6). This reflects the actual soil fertility status of the study area at the time of sampling, rather than a limitation of the methodology. No sampled locations exhibited characteristics qualifying them for high or very high fertility classes (fertility value > 75), emphasizing the need for targeted soil improvement measures and precision nutrient management.

**Table 3:** Values of soil fertility and the corresponding fertility class.

Fertility class	Fertility value
Very low (VL)	0–25
Low (L)	25–50
Moderate (M)	50–75
High (H)	75–90
Very high (VH)	90–100

**Table 4:** Descriptive statistics of measured soil properties.

Parameters	Mean	Median	StdDev	SC
N (kg/ha)	117.80	124.92	35.14	− 0.52
P (kg/ha)	28.77	29.00	7.09	− 0.07
K(kg/ha)	135.21	135.50	27.71	0.02
S (mg/kg)	14.52	15.77	6.51	− 0.35
Zn (mg/kg)	0.86	0.82	0.17	1.09
Cu (mg/kg)	0.52	0.52	0.31	0.16
Fe (mg/kg)	3.06	3.60	1.91	− 0.06
Mn (mg/kg)	2.35	2.37	0.29	0.0025
B (mg/kg)	0.44	0.44	0.11	0.2637

*Std* standard deviation, *SC* Skewness coefficient.

The spatial variability of soil fertility was classified into low, and medium fertility zones based on the FIS and kriging interpolation, covering respective proportions of the total area. The majority of the zones were classified under low fertility according to the fertility class. To enhance clarity and facilitate effective management and future applications, the low-fertility zones were further subdivided into five distinct color-coded categories. This classification allows for better visualization, targeted soil improvement strategies, and streamlined decision-making for future interventions. The dark purple (4.78%) regions indicate very low fertility, characterized by nutrient deficiency and limited crop productivity (Smith et al., 2022). The light purple (19.90%) areas represent low fertility, requiring balanced fertilization and organic amendments to enhance productivity^10,69^. The light blue (24.23%) regions also fall under the low fertility class, where soil organic matter and micronutrient management are crucial for sustaining crop growth. The greenish yellow (24.28%) zones exhibit moderate fertility levels, indicating the presence of adequate nutrients but with scope for further soil health improvement. The yellow (15.85%) category shows higher nutrient availability, suitable for crop cultivation but may require site-specific nutrient management. The red (10.97%) areas represent moderate fertility, where optimal conditions exist for most crops with minimal nutrient intervention. This classification enables targeted soil management practices, improving precision agriculture and sustainable land use^70^. The study demonstrated a strong correlation between soil fertility and crop yield, with R^2^ values of 0.919 for pearl millet, 0.890 for mustard, 0.863 for wheat, and 0.861 for barley (Fig. 9). These findings highlight the critical role of soil fertility in optimizing productivity across all crops^71,72^. The strong correlations validate the effectiveness of soil fertility-based zoning and emphasize the need for precision nutrient management to maximize yields. Implementing site-specific fertilization strategies can enhance soil health, ensuring sustainable and efficient crop production.

#### Comparison of grid-based and spatially contiguous fertility zones

**Fig. 10:**
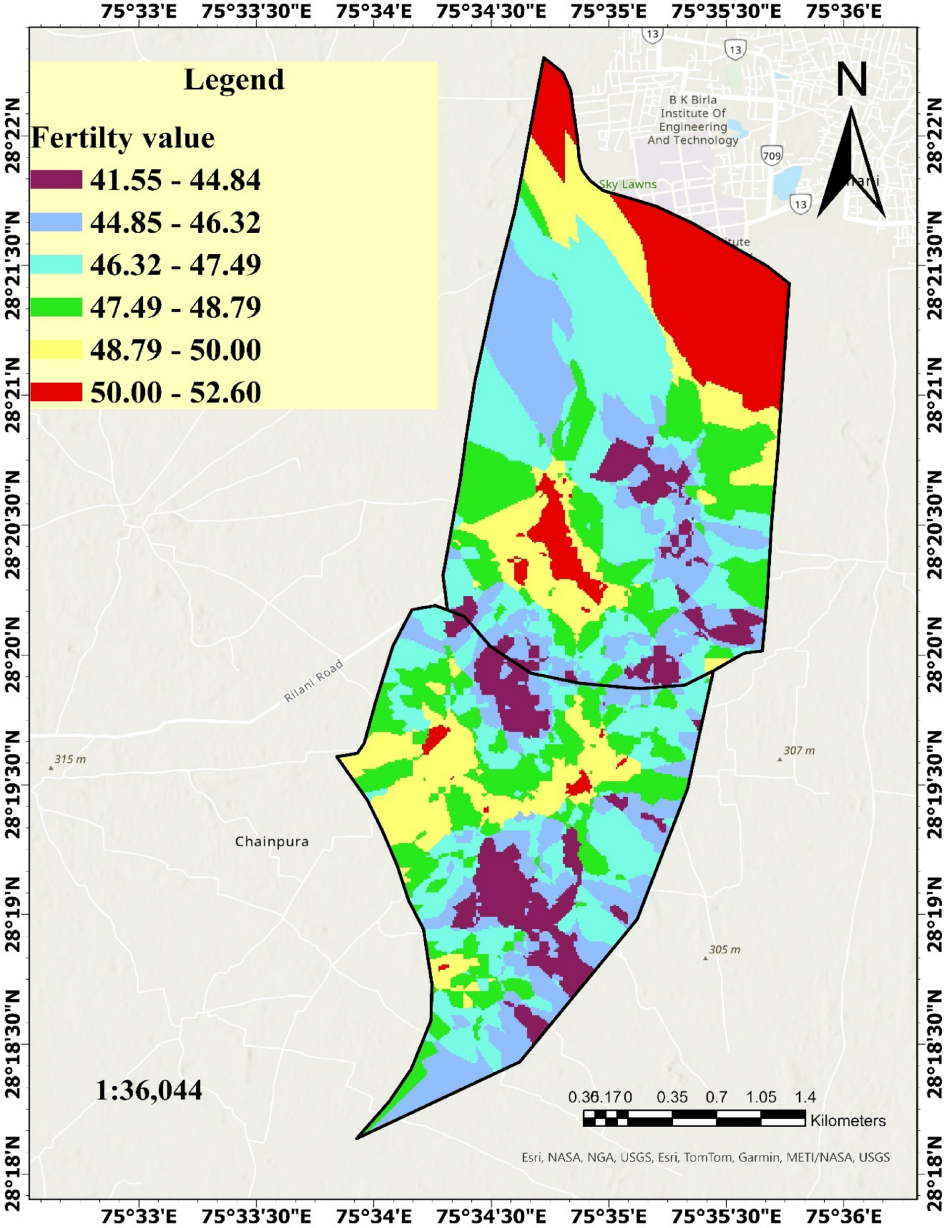
Spatially Continuous Fertility Zones using fuzzy inference system and kriging interpolation technique.

To evaluate the effect of spatial constraints on fertility zone delineation, the original fuzzy grid-based fertility map was compared with a spatially contiguous map generated using Iso Cluster Unsupervised Classification and Region Grouping, applying a minimum class size of 20 and sample interval of 10. While the grid-based map was derived directly from detailed soil sampling and preserved fine-scale variability, it resulted in fragmented spatial patterns with scattered zones that may be impractical for on-ground management applications. In contrast, the spatially constrained map produced well-defined, contiguous zones that are easier to interpret and implement at the farm level (Fig. 10). Quantitative comparison showed that although class-wise area proportions were largely preserved, minor redistributions occurred. For example, light purple areas increased from 19.90 to 25.43%, while greenish yellow areas decreased slightly due to grids grouping. These differences reflect improved internal homogeneity and spatial coherence across management units^73,74^. Despite these refinements, the original grid-based zonation remains scientifically valid, as it is rooted in systematic sampling and high-resolution fertility modeling. However, for enhanced usability and practical decision-making, the contiguous zoning approach offers advantages in field operations, extension planning, and farmer engagement^71^. This dual comparison highlights that while grid-based models provide precision, spatial smoothing enhances clarity, scalability, and adoption in real-world agricultural contexts.

### Performance assessment of fuzzy inference system

**Fig. 11:**
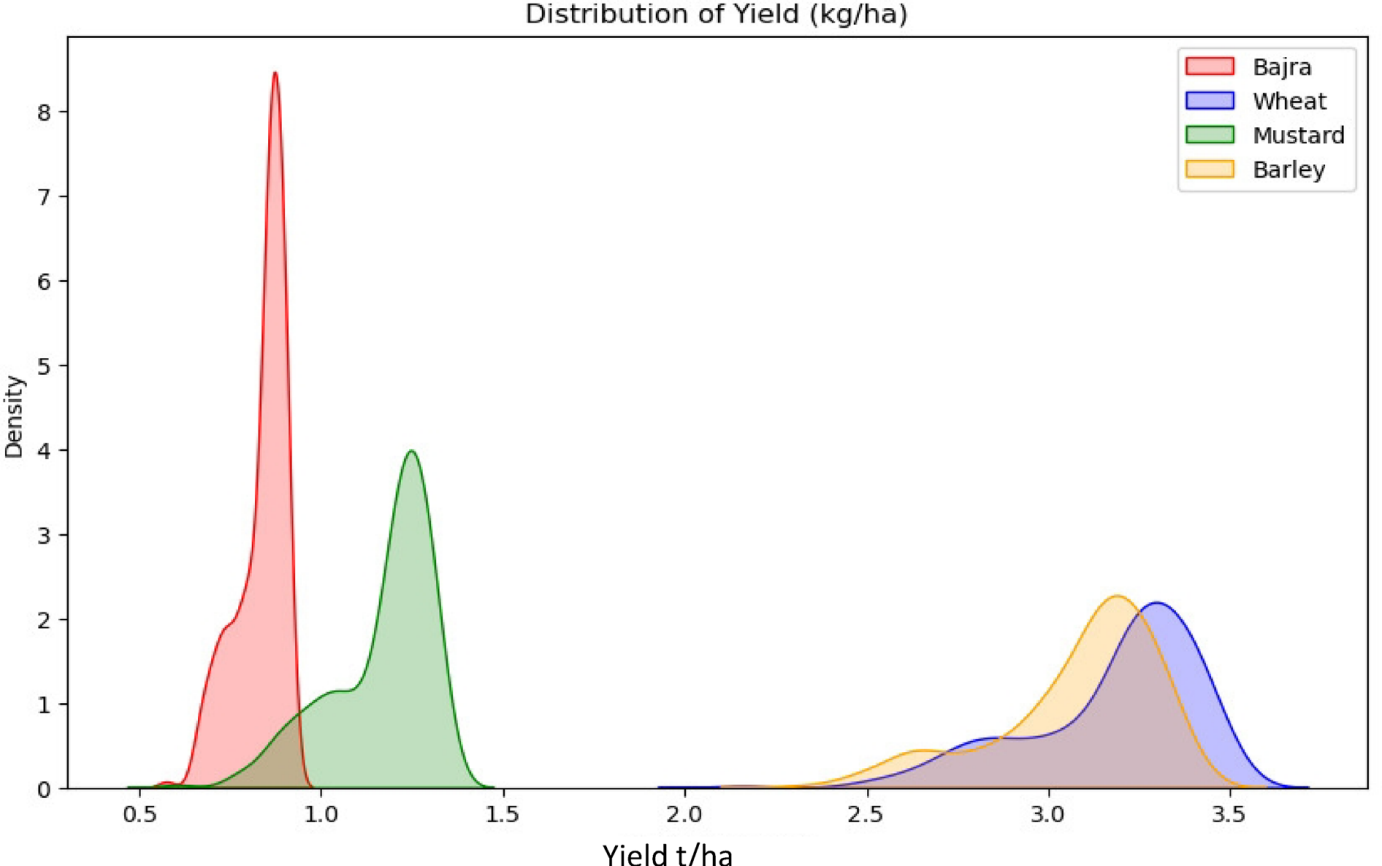
Yield distribution of selected crops (t/ha).

**Fig. 12:**
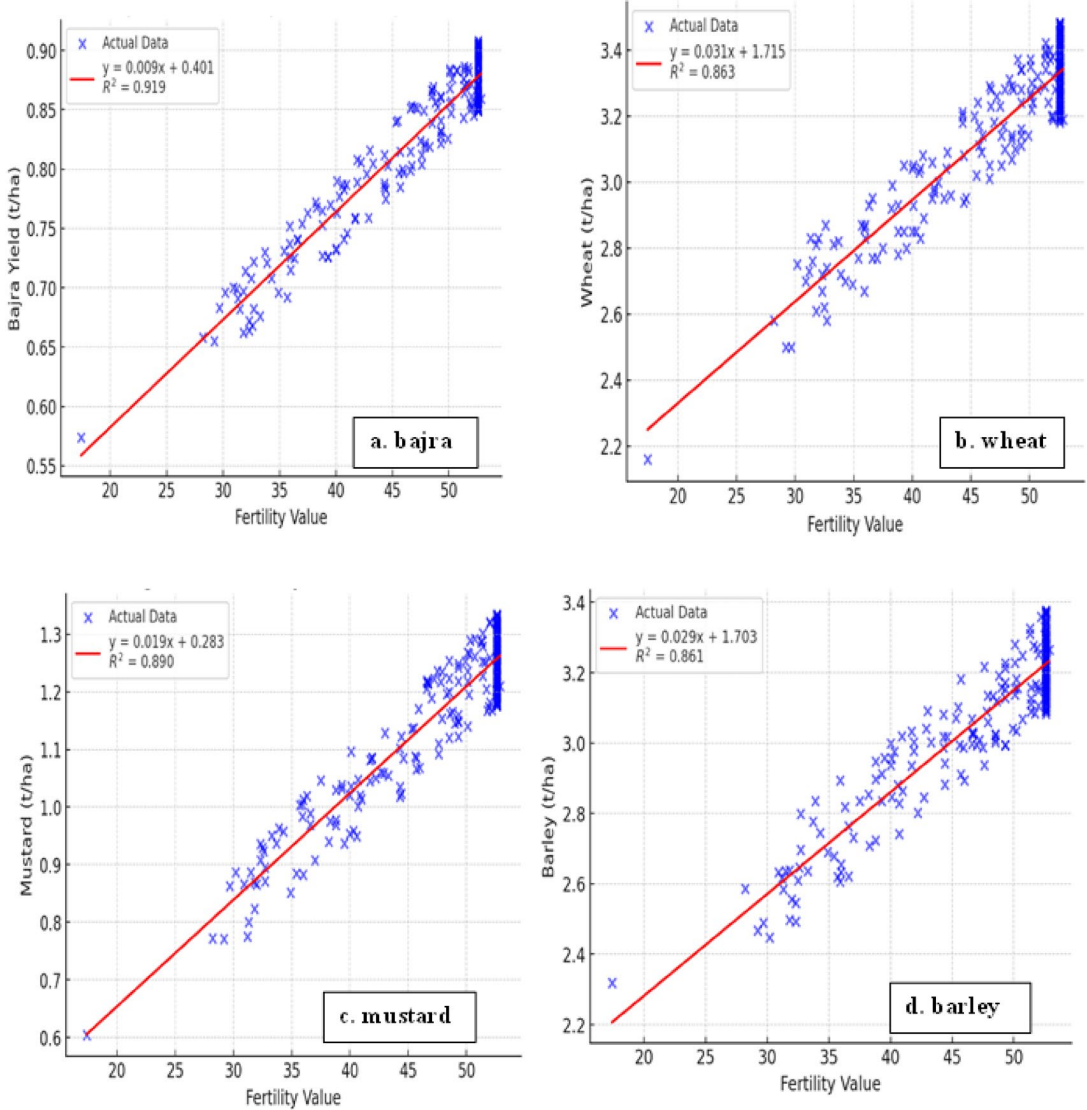
(**a**–**d**) Correlation analysis: Fertility value and observed yield of selected crops.

The predictive performance of the FIS for soil fertility assessment and crop yield estimation is illustrated in Fig. 11. The yield data were collected from the same locations as soil sampling points to establish a relationship between soil fertility and crop productivity. To evaluate whether significant differences in crop yield exist across fertility zones, Welch’s ANOVA was conducted separately for each crop. This method was selected over classical ANOVA due to unequal variances and sample sizes among fertility classes^75^. The results revealed statistically significant differences in yields for Wheat, Mustard, Barley, and Bajra across fertility classes (*p* < 0.001 for all), with large effect sizes (partial η^2^ > 0.59). Games-Howell post-hoc tests confirmed that yields in Moderate and Low fertility classes were significantly higher than those in Very Low fertility zones. The distribution of crop yields (t/ha), as shown in Fig. 11, reveals that Wheat achieves the highest productivity, followed by Mustard and Barley, while Bajra demonstrates relatively lower yield performance Regression analysis was conducted between FIS-generated fertility indices and observed crop yields to evaluate the accuracy and reliability of the model. This approach enables a quantitative assessment of FIS performance in predicting crop yield variations and supports its applicability in precision agriculture and site-specific nutrient management. Regression analysis between the soil fertility index derived from the FIS and the observed yield of barley demonstrated a strong correlation, with an R^2^ value of 0.919 in the linear regression model (Fig. 12d). Recent studies have reported strong correlations between soil fertility indices and barley yield, demonstrating that targeted soil fertility management can optimize crop performance^76,77^. Similar to that of mustard, the prediction of soil fertility status by the FIS with an observed yield of wheat were 0.890 and 0.863, respectively (Fig. 12b,c). For pearl millets, FIS showed a high correlation (R^2^ = 0.919) between soil fertility indices and observed yield, reinforcing its predictive accuracy (Fig. 12a). The strong association suggests that FIS effectively captures soil-nutrient interactions in pear millets (bajra), optimizing nutrient management for yield enhancement. Studies have shown that fuzzy logic models significantly improve soil fertility assessment and productivity predictions in dryland crops, supporting these findings^78,79^.

### Potential fertilizer savings through fertility zonation

The delineation of macro- and micronutrient-based fertility zones enables targeted nutrient application, which has the potential to reduce fertilizer use by approximately 15–30% compared to blanket application rates, depending on crop type and local variability. This estimate aligns with findings from precision agriculture studies in similar agro-ecological conditions. Such savings not only reduce input costs for farmers but also contribute to minimizing nutrient leaching and environmental impact. Future field validation will focus on quantifying exact fertilizer savings and yield improvements across different crops and management zones.

### Comparison with international case studies, limitations, and future research

In this study, fuzzy-GIS based soil fertility zonation approach aligns with internationally published studies that integrate fuzzy logic and geostatistics for soil and crop management. For instance, Seyedmohammadi et al.^42^ successfully combined fuzzy logic, kriging, and GIS to model soil texture variability, demonstrating the value of hybrid models in capturing soil heterogeneity. Similarly, Lanki et al.^41^ highlighted the global applications of GIS for soil fertility management, emphasizing the role of spatial analysis in precision agriculture. This study advances these approaches by incorporating both macro- and micronutrients for multi-crop fertility zonation, providing a more comprehensive framework for site-specific nutrient management.

In addition, Khan^80^ applied geostatistical and fuzzy clustering methods for delineating site-specific management zones in hot arid regions of India, which parallels our integration of fuzzy logic with GIS-based interpolation. Jena et al.^71^ used terrain and soil parameters to develop crop management zones through geospatial modeling, reinforcing the importance of integrated spatial datasets. Lal et al.^81^ used multi-temporal NDVI and edaphic data to define management zones, underscoring the benefit of incorporating remote sensing and temporal variability. These studies validate the effectiveness of combining fuzzy modeling with spatial data for practical, data-driven soil and crop management.

While the proposed method demonstrated strong correlation with crop yield and practical utility, certain limitations remain. The accuracy of fertility maps depends on sampling density; sparse areas may introduce interpolation uncertainty. The fuzzy rules and membership functions were based on expert knowledge and literature, which could benefit from further calibration using multi-season field data. Furthermore, the produced fertility zones were designed not only as scientific outputs but also as operational guidance for site-specific nutrient management. Efforts were made to align zonation with practical field conditions through expert validation and correlation with actual yield data. To further enhance usability for farmers, we have planned on-farm validation trials and are working towards aggregation of fuzzy zones into management units compatible with standard farm operations. Additionally, development of mobile-based decision-support tools is underway to facilitate adoption and real-time use of the zonation maps by farmers. While our study successfully produced soil fertility zones using fuzzy logic and geostatistical methods in ArcGIS Pro, cluster validity metrics such as Fuzzy Performance Index (FPI), Normalized Classification Entropy (NCE), or Multiscale Permutation Entropy (MPE) were not applied during the initial analysis because our approach was rule-based rather than clustering-based. We acknowledge this as a methodological distinction and a limitation, and we recognize that integrating these metrics would strengthen scientific rigor in studies employing fuzzy clustering. This will be considered in future research where clustering techniques are applied. Future research should focus on refining the fuzzy rule base, integrating climatic and remote sensing variables, and developing mobile decision-support tools for wider adoption across diverse agro-ecological zones.

## Conclusions

This study demonstrated that the integration of a Fuzzy Inference System (FIS) with GIS-based kriging interpolation provides an effective approach for soil fertility zonation and site-specific nutrient management in semi-arid regions. The methodological novelty of this study resides in the integrative architecture of fuzzy expert systems and geoinformatics to simultaneously model twelve macro- and micronutrients, thereby quantifying multi-element stoichiometric constraints on crop productivity that single-factor approaches overlook. The rule based fuzzy inference system has been constructed using diverse membership functions (triangular, trapezoidal, Gaussian, and S-shaped) to capture maximum and different types of uncertainties associated with variables. These variables are transformed into a composite fertility index, which is then spatially generalized via ordinary kriging (spherical variogram, 10 m grid). This geo-statistical upscaling yields spatially exhaustive fertility surfaces whose accuracy is verified against multi-crop yield observations, producing robust index–yield relationships (R^2^ = 0.86–0.92, RMSE ≤ 0.34 t ha^−1^). Scenario analysis demonstrated that zone-specific nutrient prescriptions derived from these maps can curtail fertilizer input by 15–30% relative to uniform recommendations, translating soil-chemical heterogeneity into tangible agronomic and economic gains. By integrating a comprehensive spectrum of nutrients, expert-driven fuzzy logic, high-resolution geostatistics, and rigorous agronomic validation within an openly replicable workflow, the study elevates fertility-zone delineation from descriptive mapping to a decision-support framework that is transferable across agro-ecological contexts.

## Data Availability

The datasets analyzed during the current study are not publicly available due to ethical concerns regarding privacy and the protection of personal data but are available from the corresponding author on reasonable request.
